# Variation in use of targeted therapies for metastatic renal cell carcinoma: Results from a Dutch population-based registry

**DOI:** 10.1186/s12885-016-2395-x

**Published:** 2016-06-11

**Authors:** S. De Groot, S. Sleijfer, W. K. Redekop, E. Oosterwijk, J. B. A. G. Haanen, L. A. L. M. Kiemeney, C. A. Uyl-de Groot

**Affiliations:** Institute of Health Policy and Management, Erasmus University Rotterdam, P.O. Box 1738, 3000 DR Rotterdam, The Netherlands; Department of Medical Oncology and Cancer Genomics Netherlands, Erasmus MC Cancer Institute, Rotterdam, The Netherlands; Department of Urology, Radboud Institute for Molecular Life Sciences, Radboud University Medical Center, Nijmegen, The Netherlands; Department of Medical Oncology, The Netherlands Cancer Institute–Antoni van Leeuwenhoek Hospital, Amsterdam, The Netherlands; Department for Health Evidence, Radboud Institute for Health Sciences, Radboud University Medical Center, Nijmegen, The Netherlands

**Keywords:** Metastatic renal cell carcinoma, Targeted therapy, Uptake and use, Overall survival, Population-based registry

## Abstract

**Background:**

For patients with metastatic renal cell carcinoma (mRCC), targeted therapies have entered the market since 2006. The aims of this study were to evaluate the uptake and use of targeted therapies for mRCC in The Netherlands, examine factors associated with the prescription of targeted therapies in daily clinical practice and study their effectiveness in terms of overall survival (OS).

**Methods:**

Two cohorts from PERCEPTION, a population-based registry of mRCC patients, were used: a 2008–2010 Cohort (*n* = 645) and a 2011–2013 Cohort (*n* = 233). Chi-squared tests for trend were used to study time trends in the use of targeted therapy. Patients were grouped based on the eligibility criteria of the SUTENT trial, the trial that led to sunitinib becoming standard of care, to investigate the use of targeted therapies amongst patients fulfilling those criteria. Multi-level logistic regression was used to identify patient subgroups that are less likely to receive targeted therapies.

**Results:**

Approximately one-third of patients fulfilling SUTENT trial eligibility criteria did not receive any targeted therapy (29 % in the 2008–2010 Cohort; 35 % in the 2011–2013 Cohort). Patients aged 65+ years were less likely to receive targeted therapy in both cohorts and different risk groups (odds ratios range between 0.84–0.92); other factors like number of metastatic sites were of influence in some subgroups. Amongst treated patients, there was a decreasing trend in sunitinib use over time (*p* = 0.0061), and an increasing trend in pazopanib use (*p* = 0.0005).

**Conclusions:**

Targeted therapies have largely replaced interferon-alfa as first-line standard of care. Nevertheless, many eligible patients in Dutch daily practice did not receive targeted therapies despite their ability to improve survival. Reasons for their apparent underutilisation should be examined more carefully.

**Electronic supplementary material:**

The online version of this article (doi:10.1186/s12885-016-2395-x) contains supplementary material, which is available to authorized users.

## Background

Kidney cancer accounts for about 3 % of all cancers with an estimated incidence of 115,200 in Europe in 2012 [1]. Renal cell carcinoma (RCC) represents 90 % of all kidney cancers [2]. The prognosis is relatively good for patients with localised disease, which can be treated with surgery, but the prognosis of patients with advanced or metastatic disease (mRCC) is poor [3].

Targeted therapies for mRCC have entered the market since 2006, sunitinib being the first. Sunitinib increased median progression-free survival (PFS) from five to 11 months [4], and overall survival (OS) from 22 to 26 months compared to interferon-alfa (IFN-a) in mRCC patients with a clear-cell histology [5]. Subsequently, it became standard of care for patients with a good or intermediate prognosis according to the Memorial Sloan Kettering Cancer Center (MSKCC) risk score [6]. Recently, the effectiveness of sunitinib was demonstrated in a broader ‘real-world’ population [7]. Bevacizumab (in combination with IFN-a) and pazopanib were added to guidelines as first-line therapies for patients with a good or intermediate prognosis in 2009 and 2010, respectively [6, 8]. For patients with a poor prognosis, temsirolimus was recommended [6] following the results of a multicentre, phase III trial in mRCC patients without any restrictions in histologic type, showing an increase in OS from seven to 11 months compared to IFN-a [9]. Furthermore, a number of second-line therapies have been added to guidelines, such as sorafenib, everolimus and axitinib [6, 10].

Obviously, full and swift implementation of guidelines into clinical practice is essential to maximise the benefits of new therapies. However, the adoption of innovations in cancer care is generally quite heterogeneous, and differs between countries, and regions within countries [11]. A study by Jonsson et al. showed widespread use of sunitinib in the eight of the countries they studied, despite small differences between countries [12]. Sorafenib was widely prescribed in France, while a very low uptake and use in the United Kingdom and the United States were found. Besides between-country variation, Jonsson et al. found within-country variation in Sweden and suggested that more detailed information is needed on the use of first- and second-line therapies, to determine the extent of potential under- and overconsumption in different regions and different patient populations [12].

The aims of this study were to evaluate the uptake and use of targeted therapies for mRCC in The Netherlands, examine factors associated with the prescription of targeted therapies in daily clinical practice and study their effectiveness in terms of OS.

## Methods

### Study population

A population-based registry (entitled PERCEPTION) was created to include patients with mRCC. The PERCEPTION registry consisted of two parts; a retrospective study and a prospective study. In the retrospective study, eligible patients were selected from the Netherlands Cancer Registry (NCR), which maintains a cancer registration database of all cancer patients in The Netherlands. Inclusion criteria for the retrospective study comprised a diagnosis of mRCC (i.e. metastases at initial presentation) of any histological subtype. Patients diagnosed from January 2008 until December 2010 in 42 of 51 hospitals (both general and academic) in four regions, covering approximately half of the country, were included. All patients were followed for a minimum of three years or until death (2008–2010 Cohort).

The prospective study was designed differently in order to measure additional aspects of the disease, such as health-related quality of life (not reported in this study). In the prospective study, patients with RCC (all stages) of any histological subtype diagnosed from 2011 until June 30^th^ 2013 in 25 of 32 hospitals (both general and academic) in three regions were included. In contrast to the 2008–2010 Cohort, this cohort also comprised patients with mRCC who were initially diagnosed with localised disease. Besides the NCR, the hospitals’ financing systems were used to select eligible patients at an early phase (for quality of life measurements). All patients were followed until the end of 2013 or until death (2011–2013 Cohort).

### Data collection

Data on baseline demographics, clinical and laboratory factors were retrospectively collected from individual patient records by using uniform case report forms to ensure consistent data collection. Furthermore, data on treatment schemes and treatment endpoints (e.g. survival) were collected. Laboratory factors, such as haemoglobin and corrected calcium levels, were standardised according to routinely used reference values. Data were collected by personnel of the NCR and data collection stopped at the end of 2013.

### Statistical analyses

To study differences in the proportion of patients receiving targeted therapy per half a year chi-squared tests were used. Exact tests were used to study possible time trends in the use of different therapies amongst treated patients. Additionally, chi-squared tests for trend were conducted.

Then, the use of targeted therapies within risk groups was studied. Risk groups were created using a slightly modified version of the MSKCC risk score [3]; a time from initial diagnosis to metastatic diagnosis of less than one year was used as a risk factor instead of a time from initial diagnosis to initiation of treatment of less than one year, since many patients in the study population did not receive any targeted therapy, thereby making it impossible to calculate the time to treatment. Additionally, the WHO performance status was used instead of Karnofsky performance status.

Furthermore, patients were grouped based on the eligibility criteria of the SUTENT trial [4], the trial that led to sunitinib becoming standard of care, to investigate the use of targeted therapies amongst patients fulfilling those criteria. Patients who had a clear-cell subtype, a WHO performance status of 0 or 1 and no brain metastases were classified as fulfilling the SUTENT trial eligibility criteria.

To identify patient subgroups that are less likely to receive targeted therapies in daily clinical practice among patients fulfilling SUTENT trial eligibility criteria, multilevel mixed-effects logistic regression was used to account for between-hospital variance. At the patient-level, patient and disease characteristics were taken into account including baseline demographics, clinical and laboratory factors [13, 14]. Backward selection was used to select the covariates for the models; any non-significant covariates were excluded from the models one at a time.

OS was calculated from the start of therapy until death from any cause or the date of last follow-up, whichever came first, using the Kaplan-Meier method. For patients not receiving any targeted therapy, OS was calculated from the date of diagnosis.

Missing data regarding baseline characteristics were handled using multiple imputations by chained equations. This method generated imputations based on a set of imputation models, one for each variable with missing values [15].

All analyses were performed separately for the 2008–2010 Cohort and the 2011–2013 Cohort, because of differences in inclusion criteria, patient selection and duration of follow-up. The significance level was set at α = 0.10. Data analyses were conducted using STATA statistical analysis software (StataCorp. 2013. *Stata Statistical Software: Release 13*. College Station, TX: StataCorp LP).

## Results

### Patient and disease characteristics of the 2008–2010 Cohort

714 patients newly diagnosed with mRCC between 2008 and 2010 were identified. Of these patients 69 were excluded (Additional file 1: Figure S1), leaving 645 patients for data analysis. These patients were uniformly distributed across the three-year period since 213 patients were diagnosed in 2008, 216 in 2009 and 216 in 2010. Median follow-up was 3.3 years (95 % C.I.: 3.2–3.6).

Table 1 shows the patient and disease characteristics for this cohort. Median age was 66 years (range 23–93) and the majority of patients was male (66 %). The distribution of patients according to the MSKCC risk score showed a high proportion of patients (58 %) with a poor prognosis (versus 42 % with an intermediate prognosis). Since all patients in the 2008–2010 Cohort presented with metastatic disease, none of them had a favourable prognosis (i.e. time from initial diagnosis was less than one year). Additional file 1: Table S1 provides the observed patient and disease characteristics (without imputations).

**Table 1:** Patient and disease characteristics 2008–2010 Cohort and 2011–2013 Cohort

	2008–2010 Cohort: mRCC at the initial diagnosis (*n* = 621)		2011–2013 Cohort: mRCC (*n* = 221)	
Sex - n (%)				
Female	213	34 %	60	27 %
Male	408	66 %	161	73 %
Median age - yr (range)	66	23–93	66	27–93
Histology - n (%)				
Clear cell	354	57 %	152	69 %
Other^a^	267	43 %	69	31 %
WHO performance status - n (%)				
0–1	430	69 %	178	81 %
2–4	191	31 %	42	19 %
Site of metastasis - n (%)				
One	206	33 %	87	39 %
more than one	415	67 %	134	61 %
Liver metastasis - n (%)				
No	509	82 %	175	79 %
Yes	112	18 %	46	21 %
Lung metastasis - n (%)				
No	173	28 %	74	33 %
Yes	448	72 %	147	67 %
Bone metastasis - n (%)				
No	393	63 %	158	71 %
Yes	228	37 %	63	29 %
Brain metastasis - n (%)				
No	571	92 %	200	90 %
Yes	50	8 %	16	7 %
Haemoglobin - n (%)				
Normal	205	33 %	85	38 %
< LLN	416	67 %	136	62 %
Neutrophil count - n (%)				
Normal	383	62 %	152	69 %
> ULN	238	38 %	69	31 %
Platelet count - n (%)				
Normal	452	73 %	159	72 %
> ULN	169	27 %	62	28 %
Albumin - n (%)				
Normal	391	63 %	130	59 %
< LLN	230	37 %	91	41 %
Corrected serum calcium - n (%)				
Normal	421	68 %	140	63 %
> ULN	200	32 %	81	37 %
Alkaline phosphatase - n (%)				
Normal	432	70 %	152	69 %
> ULN	189	30 %	69	31 %
Lactate dehydrogenase - n (%)				
Normal	372	60 %	179	81 %
>1.5 times ULN	249	40 %	42	19 %
Comorbidities - n (%)				
0–1	356	57 %	151	68 %
>1	265	43 %	67	30 %
Time since RCC diagnosis				
> one year	NA	NA	16	7 %
< one year	NA	NA	204	92 %

NOTE: 24 patients in the 2008–2010 Cohort and 12 patients in the 2010–2013 Cohort were excluded from this table, since these patients received a metastasectomy (combined with a nephrectomy) with a possible curative intention, making systemic treatment redundant

*Abbreviations: LLN* lower limit of normal, *ULN* upper limit of normal, *NA* not applicable

^a^mRCC was clinically established without histopathological confirmation in 17 % of patients and mRCC was classified as not otherwise specified without further subtyping in 13 % of patients (Cohort 2008–2010). It is likely that a substantial proportion of these patients had a clear cell subtype

### Uptake of targeted therapies and their use in daily clinical practice (2008–2010 Cohort)

**Fig. 1:**
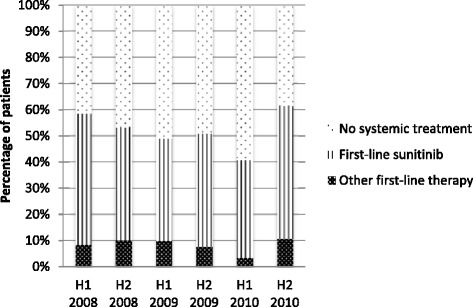
Use of first-line drugs over time per half a year (2008–2010 Cohort)

Table 2 shows the first-line therapies used in the 2008–2010 Cohort. 336/645 patients (52 %) received a first-line therapy with the majority (282, 84 %) treated with sunitinib. The distribution of patients across first-line therapies (per half-year period) is presented in Fig. 1. There is evidence of a difference between the half-year periods in the proportion of patients receiving targeted therapy (*p* = 0.041), but the chi-squared test for trend did not yield a significant result. Furthermore, no shift was found in the use of first-line therapies amongst treated patients.

**Table 2:** Treatment patterns 2008–2010 Cohort and 2011–2013 Cohort

	2008–2010 Cohort: mRCC at the initial diagnosis						2011–2013 Cohort: mRCC					
	All patients (*n* = 645)		Intermediate prognosis (*n* = 269)		Poor prognosis (*n* = 376)		All patients (*n* = 233)		Favourable/intermediate prognosis (*n* = 136)		Poor prognosis (*n* = 97)	
No systemic therapy	309 (48 %)		105 (39 %)		204 (54 %)		94 (40 %)		52 (38 %)		42 (43 %)	
First-line therapy	336 (52 %)	336 (100 %)	164 (61 %)	164 (100 %)	172 (46 %)	172 (100 %)	139 (60 %)	139 (100 %)	84 (62 %)	84 (100 %)	55 (57 %)	55 (100 %)
Sunitinib		282 (84 %)		145 (88 %)		137 (80 %)		110 (79 %)		66 (79 %)		44 (80 %)
Temsirolimus		24 (7 %)		5 (3 %)		19 (11 %)		3 (2 %)		1 (1 %)		2 (4 %)
Sorafenib		11 (3 %)		7 (4 %)		4 (2 %)		4 (3 %)		3 (4 %)		1 (2 %)
Bevacizumab + IFN-a		6 (2 %)		2 (1 %)		4 (2 %)		2 (1 %)		1 (1 %)		1 (2 %)
Pazopanib		4 (2 %)		4 (2 %)		0 (0 %)		11 (8 %)		7 (8 %)		4 (7 %)
IFN-a		3 (1 %)		0 (0 %)		3 (2 %)		1 (1 %)		0 (0 %)		1 (2 %)
Everolimus		3 (1 %)		1 (1 %)		2 (1 %)		2 (1 %)		1 (1 %)		1 (2 %)
Pazopanib-everolimus		0 (0 %)		0 (0 %)		0 (0 %)		3 (2 %)		3 (4 %)		0 (0 %)
Other		3 (1 %)		0 (0 %)		3 (2 %)		3 (2 %)		2 (2 %)		1 (2 %)
Second-line therapy	101 (16 %)	101 (100 %)	57 (21 %)	57 (100 %)	44 (12 %)	44 (100 %)	37 (16 %)	37 (100 %)	25 (18 %)	25 (100 %)	12 (12 %)	12 (100 %)
Everolimus		40 (40 %)		26 (46 %)		14 (32 %)		21 (57 %)		12 (50 %)		9 (75 %)
Sorafenib		28 (28 %)		15 (26 %)		13 (30 %)		5 (14 %)		4 (16 %)		1 (8 %)
Sunitinib		14 (14 %)		8 (14 %)		6 (14 %)		1 (3 %)		1 (4 %)		0 (0 %)
Temsirolimus		11 (11 %)		4 (7 %)		7 (16 %)		3 (8 %)		2 (8 %)		1 (8 %)
Pazopanib		4 (4 %)		2 (4 %)		2 (5 %)		5 (14 %)		4 (16 %)		1 (8 %)
Bevacizumab + IFN-a		1 (1 %)		1 (2 %)		0 (0 %)		2 (5 %)		2 (8 %)		0 (0 %)
Other		3 (3 %)		1 (2 %)		2 (5 %)		0 (0 %)		0 (0 %)		0 (0 %)

Of the 336 patients receiving first-line therapy, 101 patients (30 %) also received a second-line therapy, with everolimus being the most common (40 %), followed by sorafenib (28 %). There was an increasing trend in everolimus use over time (*p* < 0.0001) and a decreasing trend in sorafenib use (*p* < 0.0001); from 2010 onwards, everolimus largely replaced sorafenib.

### Use of targeted therapies amongst patients with an intermediate prognosis (2008–2010 Cohort)

Forty-two percent (269/645) of the patients in the 2008–2010 Cohort had an intermediate prognosis.

105/269 patients (39 %) received no targeted therapy. Some (*n* = 15) of these patients received a metastasectomy (combined with a nephrectomy) with a possible curative intention, making systemic therapy redundant. 40 of the remaining 90 patients (44 %) who were given neither targeted therapy nor a metastasectomy (combined with a nephrectomy) fulfilled the SUTENT trial eligibility criteria, indicating that they might have been eligible for treatment with sunitinib or another targeted therapy. 164/269 patients (61 %) received a first-line treatment; the majority was treated with sunitinib (145/164; 88 %). Of the 145 patients treated with sunitinib, 102 fulfilled the SUTENT trial eligibility criteria.

In patients fulfilling SUTENT trial eligibility criteria (including patients not receiving any targeted therapy and patients treated with sunitinib), patients with an abnormal neutrophil count (OR, 0.28; *p* = 0.045) were less likely to receive sunitinib, whereas patients with more than one metastatic site (OR, 3.35; *p* = 0.010) were more likely to receive sunitinib after adjustment for additional patient and disease characteristics (see frequencies in Table 3).

**Table 3:** Patient subgroups that are more of less likely to receive targeted therapy while fulfilling SUTENT trial eligibility criteria

	2008–2010 Cohort: mRCC at the initial diagnosis				2011–2013 Cohort: mRCC			
	Intermediate prognosis (*n* = 142)		Poor prognosis (*n* = 99)		Favourable/intermediate prognosis (*n* = 70)		Poor prognosis (*n* = 39)	
	No targeted therapy (*n* = 40)	Sunitinib (*n* = 102)	No targeted therapy (*n* = 29)	Sunitinib (*n* = 70)	No targeted therapy *n* = 25	Sunitinib *n* = 45	No targeted therapy *n* = 13	Sunitinib *n* = 26
Sex – n (%)								
Female	NS	NS	NS	NS	2 (8 %)	15 (33 %)	NS	NS
Male	NS	NS	NS	NS	23 (92 %)	30 (67 %)	NS	NS
Median age – yr (range)			71 (43–84)	62 (23–89)	71 (44–79)	61 (39–79)	72 (57–82)	63 (42–79)
Site of metastasis – n (%)								
one	25 (62 %)	38 (37 %)	15 (53 %)	20 (29 %)	NS	NS	NS	NS
more than one	15 (38 %)	64 (63 %)	14 (47 %)	50 (71 %)	NS	NS	NS	NS
Neutrophil count – n (%)								
normal	27 (68 %)	87 (85 %)	NS	NS	NS	NS	NS	NS
> ULN	13 (33 %)	15 (15 %)	NS	NS	NS	NS	NS	NS
Comorbidities								
Zero or one	NS	NS	15 (52 %)	52 (74 %)	NS	NS	NS	NS
More than one	NS	NS	14 (48 %)	18 (26 %)	NS	NS	NS	NS

NOTE: This table shows patient subgroups that are more or less likely to receive targeted therapy (i.e. first-line sunitinib) among patients fulfilling SUTENT trial eligibility criteria (according to the multi-level mixed-effects models). The multi-level models initially included all patient and disease characteristics as mentioned in Table 1 (besides hospital of diagnosis). Not significant (NS) means that this variable was not significantly associated to prescription of sunitinib at α = 0.10 in a particular risk group/cohort

*Abbreviations*: *NS* not significant

The median OS of eligible patients not receiving any targeted therapy was 18.6 months (95 % C.I. 8.4–33.7). Table 4 presents the median OS in subgroups of patients with an intermediate prognosis treated with first-line sunitinib. Median OS of eligible patients treated with sunitinib was 14.8 months (95 % C.I. 10.8–16.1). Note that a different starting point was used for the survival analysis (compared to the survival analysis in patients not receiving any targeted therapy). The mean time from diagnosis to start of first-line sunitinib was 4.3 months (standard deviation [SD] 6.0).

**Table 4:** Overall survival in subgroups of patients treated with first-line sunitinib (Cohort 2008–2010 and Cohort 2011–2013)

		2008–2010 Cohort: mRCC at the initial diagnosis			2011–2013 Cohort: mRCC		
		n	Median OS in months (95 % C.I.)	p-value	n	Median OS in months (95 % C.I.)	p-value
All patients		282	9.1 (7.2–11.1)	–	109	10.1 (7.2–13.8)	–
Fulfilling SUTENT trial eligibility criteria	No	110	6.5 (4.9–8.9)		38	6.9 (3.4–10.9)	
	Yes	172	11.9 (8.8–14.6)	0.0014	71	12.1 (8.9-NR)	0.0074
Brain metastases	No	261	9.3 (7.6–11.9)		101	10.9 (7.8–18.0)	
	Yes	21	4.3 (2.1–11.5)	0.0820	8	2.5 (0.8–7.5)	0.0125
WHO performance status	0–1	248	10.3 (8.4–13.0)		100	11.3 (7.8–18.0)	
	2–4	34	3.3 (1.8–6.2)	<0.0001	9	1.4 (0.6–7.5)	<0.0001
Histology	Clear cell	204	10.0 (7.6–13.3)		81	10.6 (7.2–20.3)	
	Non-clear cell	78	6.9 (5.4–11.0)	0.0809	28	10.0 (3.5–13.8)	0.3325
Age	<65 years	162	8.9 (6.5–10.8)		64	11.3 (7.2–20.3)	
	> = 65 years	120	10.0 (6.5–13.8)	0.8373	45	10.0 (5.3–16.6)	0.4294
Patients with an intermediate prognosis (or favourable prognosis)^a^		145	14.6 (11.5–16.0)	–	65	16.6 (10.1-NR)	–
Fulfilling SUTENT trial eligibility criteria	No	43	11.9 (6.5–18.3)		20	10.9 (2.7-NR)	
	Yes	102	14.8 (10.8–16.1)	0.2897	45	18.0 (10.1-NR)	0.1212
Brain metastases	No	136	14.6 (10.7–16.0)		61	16.6 (10.9-NR)	
	Yes	9	11.9 (4.3–29.3)	0.8072	4	6.9 (2.5-NR)	0.2282
WHO performance status	0–1	143	14.4 (10.8–16.0)		64	16.6 (10.1-NR)	
	2–4	2	–	0.2304	1	–	0.2471
Histology	Clear cell	111	14.8 (11.8–16.2)		49	18.0 (10.0-NR)	
	Non-clear cell	34	11.5 (6.3–17.7)	0.1954	16	13.8 (2.7-NR)	0.3135
Age	<65 years	87	10.8 (7.2–15.7)		36	12.1 (7.2-NR)	
	> = 65 years	58	16.1 (12.4–18.8)	0.2606	29	16.6 (8.5-NR)	0.7157
Patients with a poor prognosis		137	6.1 (4.9–7.7)	–	44	6.5 (3.4–10.0)	–
Fulfilling SUTENT trial eligibility criteria	No	67	4.7 (3.3–6.9)		18	3.5 (1.3–7.8)	
	Yes	70	6.8 (5.3–10.7)	0.0145	26	6.6 (3.8-NR)	0.0720
Brain metastases	No	125	6.5 (5.3–8.4)		40	6.5 (3.8–10.1)	
	Yes	12	2.1 (0.7–4.2)	0.0062	4	1.2 (0.8-NR)	0.0134
WHO performance status	0–1	105	6.9 (5.3–9.8)		36	6.6 (3.8–10.1)	
	2–4	32	3.1 (1.4–5.5)	<0.0001	8	1.2 (0.6–7.5)	0.0087
Histology	Clear cell	93	6.1 (4.6–7.8)		32	6.5 (2.7–10.1)	
	Non-clear cell	44	5.7 (3.7–10.3)	0.6585	12	4.1 (2.6-NR)	0.9982
Age	<65 years	75	6.9 (4.9–9.8)		28	7.8 (3.8–13.7)	
	> = 65 years	62	5.4 (3.8–6.8)	0.4044	16	3.2 (1.1–6.6)	0.0256

*Abbreviations*: *C.I*, confidence interval, *NR* not reached

^a^Since all patients in the 2008–2010 Cohort presented with metastatic disease, none of the patients had a favourable prognosis (i.e. time from initial RCC diagnosis was less than one year)

Median OS was 11.9 months (95 % C.I. 6.5–18.3) for ineligible patients treated with sunitinib, which was not significantly shorter than the OS of eligible patients treated with sunitinib. No significant differences were observed within the other subgroups.

### Use of targeted therapies amongst patients with a poor prognosis (2008–2010 Cohort)

Fifty-eight percent (376/645) of the patients in the 2008–2010 Cohort, had a poor prognosis. 204/376 patients (54 %) did not receive any targeted therapy. Of these patients, 9 patients received a metastasectomy (combined with a nephrectomy). 29 of the remaining 195 patients (15 %) who were given neither targeted therapy nor a metastasectomy (combined with a nephrectomy) fulfilled the SUTENT trial eligibility criteria. 172/376 (46 %) patients received a first-line treatment, which was mainly sunitinib (137/376; 80 %). Of the 137 patients treated with sunitinib, 70 fulfilled the SUTENT trial eligibility criteria.

Amongst patients fulfilling SUTENT trial eligibility criteria, older patients (OR, 0.90; *p* = 0.006) and patients with more than one comorbidity (OR, 0.26; *p* = 0.090) were less likely to receive sunitinib, whereas patients with more than one metastatic site (OR, 5.38; *p* = 0.034) were more likely to receive sunitinib (see frequencies in Table 3). Furthermore, a significant association was found between hospital of diagnosis and prescription of sunitinib (*p* = 0.0059).

Median OS of eligible patients not receiving any targeted therapy was 6.2 months (95 % C.I. 1.7–9.9). Table 4 shows the median OS in subgroups of patients with a poor prognosis treated with first-line sunitinib. Median OS of eligible patients treated with sunitinib was 6.8 months (95 % C.I. 5.3–10.7). The mean time from diagnosis to start of first-line sunitinib was 2.9 months (SD 5.5).

Median OS was significantly reduced in poor-prognosis patients treated with sunitinib but not fulfilling the SUTENT trial eligibility criteria (4.7 months, 95 % C.I. 3.3–6.9). Additionally, OS was significantly reduced in patients with brain metastases and patients with a WHO performance status of 2–4.

### Patient and disease characteristics of the 2011–2013 Cohort

The second cohort study included 791 patients with (m)RCC diagnosed between 2011 and 2013. Of these patients, 233 had metastatic disease; 75 in 2011, 102 in 2012 and 55 in 2013 (one unknown). Median follow-up of the patients with mRCC was 1.2 years (95 % C.I. 1.1–1.4).

Table 1 shows the patient and disease characteristics of the patients with mRCC in this cohort. Median age was 66 years, and 73 % (170/233) of the patients was men. Metastatic disease was present in 77 % (179/233) of patients at the time of diagnosis, whereas 23 % was initially diagnosed with localised disease. In this cohort, 4 % of the patients with mRCC had a favourable prognosis, whereas 54 % and 42 % had an intermediate or poor prognosis, respectively.

### Uptake of targeted therapies and their use in daily clinical practice (2011–2013 Cohort)

**Fig. 2:**
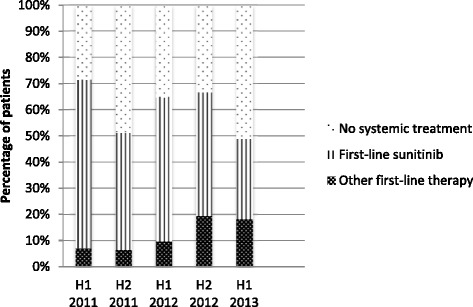
Use of first-line drugs over time per half a year (2011–2013 Cohort)

Table 2 shows the first-line therapies used in the 2011–2013 Cohort. During the follow-up period, 139/233 (60 %) patients received a first-line therapy; the majority (110, 79 %) was treated with sunitinib. The distribution of patients across first-line therapies over time (half-year periods) is presented in Fig. 2. There were no significant differences between the half-year periods in the proportion of patients receiving targeted therapies. However, amongst treated patients, there was a decreasing trend in sunitinib use over time (*p* = 0.0061) and an increasing trend in pazopanib use (*p* = 0.0005).

Thirty-seven patients also received a second-line therapy within the follow-up period. The majority was treated with everolimus (57 %), but a decreasing trend in everolimus use over time was observed (*p* = 0.0020).

### Use of targeted therapies amongst patients with a favourable or intermediate prognosis (2011–2013 Cohort)

136/233 patients (58 %) had a favourable or intermediate prognosis. 52/136 patients (38 %) did not receive any targeted therapy within the follow-up period. However, 12 of these 52 patients received a metastasectomy (combined with a nephrectomy). 25 of the remaining 40 patients (63 %) who were given neither targeted therapy nor a metastasectomy (combined with a nephrectomy) fulfilled the SUTENT trial eligibility criteria. In addition, 45 of the 66 patients treated with sunitinib fulfilled the SUTENT trial eligibility criteria.

Amongst patients fulfilling SUTENT trial eligibility criteria, males (OR, 0.12; *p* = 0.020) and older patients (OR, 0.92; *p* = 0.011) were less likely to receive sunitinib after adjustment for additional patient and disease characteristics (see frequencies in Table 3).

Median OS of eligible patients not receiving any targeted therapy was 20.9 months (95 % C.I. 7.4-not reached [NR]). Table 4 presents the median OS in subgroups of patients with a favourable or intermediate prognosis treated with first-line sunitinib. Median OS of eligible patients treated with sunitinib was 18.0 months (95 % C.I. 10.1-NR). The mean time from diagnosis to start of first-line sunitinib was 2.1 months (SD 3.3).

Median OS was 10.9 months (95 % C.I. 2.7-NR) for patients treated with sunitinib but not fulfilling SUTENT trial eligibility criteria. No significant differences were observed within subgroups.

### Use of targeted therapies amongst patients with a poor prognosis (2011–2013 Cohort)

97/233 patients (42 %) had a poor prognosis. Forty-two patients (43 %) did not receive any targeted therapy; thirteen of these 42 patients (31 %) fulfilled the SUTENT trial eligibility criteria. Of the 44 patients treated with sunitinib, 26 fulfilled the SUTENT trial eligibility criteria.

Of patients fulfilling SUTENT trial eligibility criteria, older patients (OR, 0.84; *p* = 0.012) were less likely to receive sunitinib (see frequencies in Table 3). The unadjusted model showed a significant association between hospital of diagnosis and the prescription of sunitinib, but this association disappeared after adjustment for demographics, clinical and laboratory factors.

Median OS of eligible patients not receiving any targeted therapy was 3.4 months (95 % C.I. 0.8- NR). Table 4 shows the median OS in subgroups of patients with a poor prognosis treated with first-line sunitinib. Median OS of eligible patients treated with sunitinib was 6.6 months (95 % C.I. 3.8-NR). The mean time from diagnosis to start of first-line sunitinib was 1.9 months (SD 1.8).

Median OS was significantly reduced in patients not fulfilling the SUTENT trial eligibility criteria (3.5 months, 95 % C.I. 1.3–7.8). Additionally, as in the 2008–2010 Cohort, median OS was significantly reduced in patients with brain metastases and patients with a WHO performance status of 2–4. OS was also significantly reduced in older patients.

## Discussion

Since 2006, several new targeted therapies for mRCC have entered the market and randomised controlled trial (RCTs) have shown that these therapies improve survival [4, 5, 9, 16–27]. This study examined the uptake and use of targeted therapies in The Netherlands. Not unexpected, targeted therapies, sunitinib in particular, have largely replaced IFN-a as first-line standard of care. Few patients were treated with bevacizumab (combined with IFN-a) or temsirolimus in the 2008–2013 period, even though these therapies were added to the ESMO guidelines in 2009 [6], and to Dutch guidelines in 2010 [6]. Pazopanib has only been recommended since 2010 [8], which partly explains why an increase in its use was only seen from 2012. Furthermore, there was a shift in the use of second-line therapies, where sorafenib was replaced by everolimus as the most frequent choice from 2010 onwards.

The median OS of patients with an intermediate prognosis treated with sunitinib in Dutch daily practice and fulfilling the SUTENT trial eligibility criteria was shorter than the median OS of patients in the SUTENT trial with an intermediate prognosis, i.e. 14.8 months (95 % C.I. 10.8–16.1) in the 2008–2010 Cohort compared to 20.7 months (95 % C.I. 18.2–25.6) in the SUTENT trial [5]. However, the difference was much smaller for the 2011–2013 Cohort (median OS, 18.0 months (95 % C.I. 10.1-NR)) compared to the SUTENT trial patients. Median OS of patients with a poor prognosis fulfilling the SUTENT trial eligibility criteria was similar to the median OS found in the SUTENT trial, i.e. 6.8 months (95 % C.I. 5.3–10.7) in the 2008–2010 Cohort and 6.6 months (95 % C.I. 3.8-NR) in the 2011–2013 Cohort compared to 5.3 months (95 % C.I. 4.2–10.0) in the SUTENT trial [5].

The median OS of patients with an intermediate prognosis treated with sunitinib in Dutch daily practice (regardless of their SUTENT trial eligibility status) was shorter than the OS in the expanded-access trial [7]. Median OS of patients with a poor prognosis was in line with the results of the expanded-access trial. The median OS of patients with an intermediate prognosis treated with sunitinib in Dutch daily practice was also shorter than the OS in a retrospective, non-interventional study in Australia [28]. These findings may indicate that the patients in the PERCEPTION registry with an intermediate risk had a worse prognosis than the patients with an intermediate risk in other studies.

While previous studies suggest that patients fulfilling SUTENT trial eligibility criteria have a survival benefit from first-line sunitinib [5], many eligible patients did not receive sunitinib (or any other targeted therapy) in daily practice. This was also seen in England where one in three patients with mRCC eligible for either sunitinib or pazopanib did not receive the drug [29]. Patients aged 65+ years were less likely to receive targeted therapy than younger patients after adjustment for other factors. This age factor was found in patients with an intermediate prognosis (2011–2013 Cohort) and in patients with a poor prognosis (2008–2010 Cohort and 2011–2013 Cohort). There are several explanations for this association, including medical contraindications, other grounds for physician reluctance, and patient refusal. Additionally, patients with one metastatic site were less likely to receive sunitinib (according to the 2008–2010 Cohort results), which might be explained by patients with low volume but unresectable metastases whose targeted therapy is delayed. Nevertheless, most of these patients died within the follow-up period without receiving targeted therapy at any point in time. The reasons for apparent underutilisation of targeted therapies should be examined more carefully. While hospital-level factors may also affect utilisation and lead to between-hospital variation, we found no significant differences in the prescription of targeted therapy between hospitals, except for the patients with a poor prognosis in the 2008–2010 Cohort. However, the sample size per hospital was small and the statistical power to show a difference was therefore limited.

Although this study mainly focussed on patients fulfilling SUTENT trial eligibility criteria, we found that many patients in daily clinical practice are different from patients included in RCTs. In the total study population, only 42 % and 58 % fulfilled the SUTENT trial eligibility criteria in the 2008–2010 Cohort and 2011–2013 Cohort, respectively. This was partly caused by the inclusion criteria of the PERCEPTION registry, which consisted of a diagnosis of mRCC (i.e. metastases at initial presentation in the 2008–2010 Cohort) of *any* histological subtype. Since many patients are excluded from clinical trials, such as patients with a non clear-cell subtype, patients with a WHO performance status of 2 to 4 and patients with brain metastases, one could argue that the results of these trials only apply to a subgroup of patients.

A limitation of this study is the amount of missing data in baseline characteristics, which is inherent to an observational study. To overcome this problem, multiple imputations by chained equations were conducted, which ensure that all patients are included in the analysis but simultaneously ensure that the uncertainties from missing data are retained [15]. Additionally, eligibility criteria, such as the presence of measurable disease and adequate organ function were not taken into account when determining whether patients fulfilled the SUTENT trial eligibility criteria, since data on these criteria were lacking in the PERCEPTION registry. As a consequence, some of the patients that we labelled as eligible in this study were not in fact eligible for targeted therapy. However, since we used WHO performance status to classify patients, and since we expect a relationship between WHO performance status and organ function, we believe that this could only have had a limited effect on our conclusions about the uptake and use of targeted therapies. Furthermore, the follow-up length of the 2011–2013 Cohort was limited. As a consequence, patients might have received targeted therapy after the follow-up period, leading to an underestimate of actual targeted therapy use. However, this limitation is only relevant for patients treated later in the 2011–2013 period who did not die. Lastly, OS was calculated from the date of diagnosis (i.e. metastatic disease) for patients not receiving any targeted therapy and from the start of therapy for patients treated with targeted therapy; as a consequence a comparison between the two is impossible. This approach was based on the one used in other studies to enable comparisons between the OS of patients treated with sunitinib in our study with the OS of patients treated with sunitinib in other studies [5, 7, 28].

## Conclusions

In conclusion, targeted therapies, sunitinib in particular, have largely replaced IFN-a as the first-line standard of care in The Netherlands. Nevertheless, many patients in Dutch daily practice fulfilling SUTENT trial eligibility criteria did not receive sunitinib (or any other targeted therapy) even though it could improve their survival. For example, older patients were less likely to receive sunitinib, perhaps because physicians are reluctant to prescribe it. The reasons for apparent underutilisation of targeted therapies should be examined more carefully.

## Abbreviations

C.I., confidence interval; IFN-a, interferon-alfa; mRCC, metastatic renal cell carcinoma; MSKCC, Memorial Sloan Kettering Cancer Center; NCR, Netherlands Cancer Registry; NR, not reached; OS, overall survival; PERCEPTION, PharmacoEconomics in Renal CEll carcinoma: a PopulaTION-based registry; PFS, progression-free survival; RCC, renal cell carcinoma; RCT, randomised controlled trial; SD, standard deviation.

## Additional file

Additional file 1: Figure S1. Patient enrolment. **Table S1.** Patient and disease characteristics (observed and imputed) 2008–2010 Cohort and 2011–2013 Cohort. (DOCX 57 kb)
