# Supplementary material for: Variation in use of targeted therapies for metastatic renal cell carcinoma: Results from a Dutch population-based registry
Source: BMC Cancer. 2016 Jun 11;16:364. doi: 10.1186/s12885-016-2395-x (PMC4902930; doi:10.1186/s12885-016-2395-x)
Supplement: Additional file 1: Figure S1. — Patient enrolment. Table S1. Patient and disease characteristics (observed and imputed) 2008–2010 Cohort and 2011–2013 Cohort. (DOCX 57 kb) [file 12885_2016_2395_MOESM1_ESM.docx]

**Additional file Figure 1. Patient enrolment**

PERCEPTION registry

Lost to follow-up (n=16)

- Patients continued (possible) systemic treatment in a non-participating hospital

Follow-up (n=807)

Analysis (n=233/791)

(Patients with metastatic disease were included in the analysis)

Follow-up (n=675)

Analysis (n=645)

Lost to follow-up (n=30)

- Patients continued treatment in a non-

participating hospital

Excluded (n=39)

- Not meeting inclusion criteria (n=26)
- No renal cell carcinoma (n=12)
- No metastases at presentation (n=6)
- < 18 years (n=7)
- Other (n=1)
- Other reasons (n=13)
- Diagnosed and/or treated in a non-participating hospital, and too little information available on patient and disease characteristics (and treatment) (n=12)
- Other (n=1)

Cohort 2011-2013

- (m)RCC
- Diagnosis 2011-2013

Cohort 2008-2010

- mRCC (i.e. metastases at initial presentation)
- Diagnosis 2008-2010

Assessed for eligibility (n=714)

**Additional file Table 1. Patient and disease characteristics (observed and imputed) 2008-2010 Cohort and 2011-2013 Cohort**

|  | 2008-2010 Cohort:  mRCC at the initial diagnosis | | | | 2011-2013 Cohort:  mRCC | | | |
| --- | --- | --- | --- | --- | --- | --- | --- | --- |
|  | Real world-data (n=621) | | Imputed data (n=621) | | Real world-data (n=221) | | Imputed data (n=221) | |
| Sex - n (%) |  |  |  |  |  |  |  |  |
| Female | 213 | 34% | 213 | 34% | 60 | 27% | 60 | 27% |
| Male | 408 | 66% | 408 | 66% | 61 | 73% | 161 | 73% |
| Median age - yr (range) | 66 | 23-93 | 66 | 23-93 | 66 | 27-93 | 66 | 27-93 |
| Histology - n (%) |  |  |  |  |  |  |  |  |
| Clear cell | 354 | 57% | 354 | 57% | 152 | 69% | 152 | 69% |
| Other * | 267 | 43% | 267 | 43% | 69 | 31% | 69 | 31% |
| WHO performance status - n (%) |  |  |  |  |  |  |  |  |
| 0-1 | 204 | 33% | 430 | 69% | 94 | 43% | 178 | 81% |
| 2-4 | 61 | 10% | 191 | 31% | 13 | 6% | 42 | 19% |
| Missing | 356 | 57% |  |  | 114 | 52% |  |  |
| Site of metastasis - n (%) |  |  |  |  |  |  |  |  |
| One | 195 | 31% | 206 | 33% | 85 | 38% | 87 | 39% |
| more than one | 398 | 64% | 415 | 67% | 131 | 59% | 134 | 61% |
| Missing | 28 | 5% |  |  | 5 | 2% |  |  |
| Liver metastasis - n (%) |  |  |  |  |  |  |  |  |
| No | 487 | 78% | 509 | 82% | 171 | 77% | 175 | 79% |
| Yes | 106 | 17% | 112 | 18% | 45 | 20% | 46 | 21% |
| Missing | 28 | 5% |  |  | 5 | 2% |  |  |
| Lung metastasis - n (%) |  |  |  |  |  |  |  |  |
| No | 163 | 26% | 173 | 28% | 72 | 33% | 74 | 33% |
| Yes | 430 | 69% | 448 | 72% | 144 | 65% | 147 | 67% |
| Missing | 28 | 5% |  |  | 5 | 2% |  |  |
| Bone metastasis - n (%) |  |  |  |  |  |  |  |  |
| No | 375 | 60% | 393 | 63% | 154 | 70% | 158 | 71% |
| Yes | 218 | 35% | 228 | 37% | 62 | 28% | 63 | 29% |
| Missing | 28 | 5% |  |  | 5 | 2% |  |  |
| Brain metastasis - n (%) |  |  |  |  |  |  |  |  |
| No | 546 | 88% | 571 | 92% | 200 | 90% | 200 | 90% |
| Yes | 47 | 8% | 50 | 8% | 16 | 7% | 16 | 7% |
| Missing | 28 | 5% |  |  | 5 | 2% |  |  |
| Haemoglobin - n (%) |  |  |  |  |  |  |  |  |
| Normal | 171 | 28% | 205 | 33% | 76 | 34% | 85 | 38% |
| < LLN | 347 | 56% | 416 | 67% | 122 | 55% | 136 | 62% |
| Missing | 103 | 17% |  |  | 23 | 10% |  |  |
| Neutrophil count - n (%) |  |  |  |  |  |  |  |  |
| Normal | 203 | 33% | 383 | 62% | 82 | 37% | 152 | 69% |
| > ULN | 108 | 17% | 238 | 38% | 41 | 19% | 69 | 31% |
| Missing | 310 | 50% |  |  | 98 | 44% |  |  |
| Platelet count - n (%) |  |  |  |  |  |  |  |  |
| Normal | 358 | 58% | 452 | 73% | 127 | 57% | 159 | 72% |
| > ULN | 140 | 23% | 169 | 27% | 51 | 23% | 62 | 28% |
| Missing | 123 | 20% |  |  | 43 | 19% |  |  |
| Albumin - n (%) |  |  |  |  |  |  |  |  |
| Normal | 247 | 40% | 391 | 63% | 86 | 39% | 130 | 59% |
| < LLN | 136 | 22% | 230 | 37% | 61 | 28% | 91 | 41% |
| Missing | 238 | 38% |  |  | 74 | 33% |  |  |
| Corrected serum calcium - n (%) |  |  |  |  |  |  |  |  |
| Normal | 243 | 39% | 421 | 68% | 88 | 40% | 140 | 63% |
| > ULN | 116 | 19% | 200 | 32% | 51 | 23% | 81 | 37% |
| Missing | 262 | 42% |  |  | 82 | 37% |  |  |
| Alkaline phosphatase - n (%) |  |  |  |  |  |  |  |  |
| Normal | 324 | 52% | 432 | 70% | 112 | 51% | 152 | 69% |
| > ULN | 139 | 22% | 189 | 30% | 48 | 22% | 69 | 31% |
| Missing | 158 | 25% |  |  | 61 | 28% |  |  |
| Lactate dehydrogenase - n (%) |  |  |  |  |  |  |  |  |
| Normal | 277 | 45% | 372 | 60% | 130 | 59% | 179 | 81% |
| > 1.5 times ULN | 174 | 28% | 249 | 40% | 31 | 14% | 42 | 19% |
| Missing | 170 | 27% |  |  | 60 | 27% |  |  |
| Comorbidities - n (%) |  |  |  |  |  |  |  |  |
| 0-1 | 356 | 57% | 356 | 57% | 151 | 68% | 151 | 68% |
| >1 | 265 | 43% | 265 | 43% | 67 | 30% | 67 | 30% |
| Missing | 0 | 0% |  |  | 3 | 1% |  |  |
| Time since RCC diagnosis - n (%) |  |  |  |  |  |  |  |  |
| > one year | NA | NA | NA | NA | 16 | 7% | 16 | 7% |
| < one year | NA | NA | NA | NA | 204 | 92% | 204 | 92% |
|  |  |  |  |  |  |  |  |  |

Abbreviations: LLN, lower limit of normal; ULN, upper limit of normal; NA, not applicable.
